# Supplementary material for: Corynebacteria of the diphtheriae Species Complex in Companion Animals: Clinical and Microbiological Characterization of 64 Cases from France
Source: Microbiol Spectr. 2023 Apr 6;11(3):e00006-23. doi: 10.1128/spectrum.00006-23 (PMC10269909; doi:10.1128/spectrum.00006-23)

**Supplementary Figure S1**. **Geographic distribution of the MLST sequence types (ST).** Colors correspond to STs, as indicated on the Minimum spanning tree and on the key. For two cases, location was not available and they are therefore not represented here.

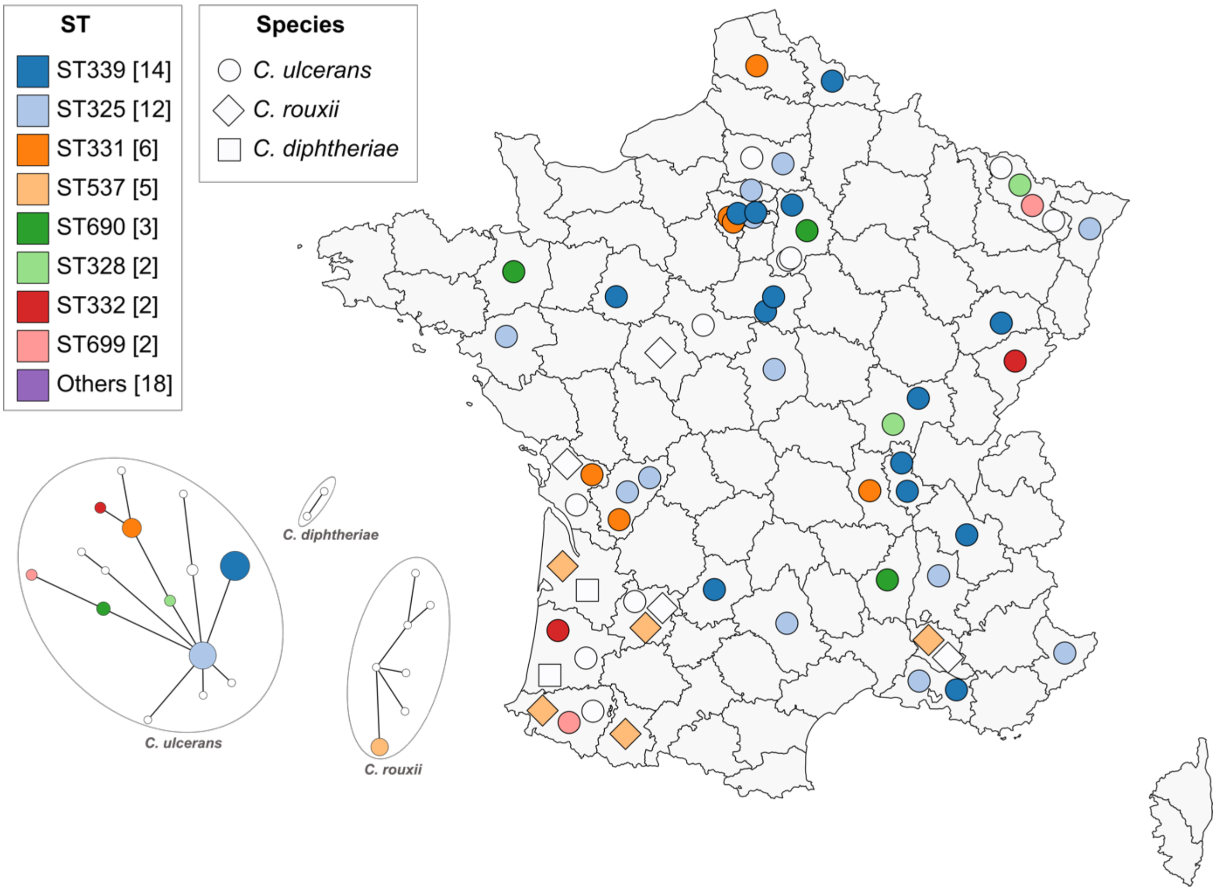

Supplement: Supplemental file 1 — Supplemental material. Download spectrum.00006-23-s0001.pdf, PDF file, 0.4 MB [file spectrum.00006-23-s0001.pdf]
